# Elevated Glucagon-like Peptide-1 Receptor Level in the Paraventricular Hypothalamic Nucleus of Type 2 Diabetes Mellitus Patients

**DOI:** 10.3390/ijms232415945

**Published:** 2022-12-15

**Authors:** Éva Renner, Fanni Dóra, Erzsébet Oszwald, Árpád Dobolyi, Miklós Palkovits

**Affiliations:** 1 Human Brain Tissue Bank, Semmelweis University, 1094 Budapest, Hungary; 2 Human Brain Tissue Bank and Microdissection Laboratory, Semmelweis University, 1094 Budapest, Hungary; 3 Laboratory of Neuromorphology, Department of Anatomy, Histology and Embryology, Semmelweis University, 1094 Budapest, Hungary; 4 Laboratory of Molecular and Systems Neurobiology, Department of Physiology and Neurobiology, Eötvös Loránd University, 1117 Budapest, Hungary

**Keywords:** glucose, food intake, hypothalamus, insulin, brain, gene expression, distribution

## Abstract

Glucagon-like peptide-1 (GLP-1) receptor (GLP-1R) agonists have been approved for the treatment of type 2 diabetes mellitus (T2DM); however, the brain actions of these drugs are not properly established. We used post mortem microdissected human hypothalamic samples for RT-qPCR and Western blotting. For in situ hybridization histochemistry and immunolabelling, parallel cryosections were prepared from the hypothalamus. We developed in situ hybridization probes for human GLP-1R and oxytocin. In addition, GLP-1 and oxytocin were visualized by immunohistochemistry. Radioactive in situ hybridization histochemistry revealed abundant GLP-1R labelling in the human paraventricular hypothalamic nucleus (PVN), particularly in its magnocellular subdivision (PVNmc). Quantitative analysis of the mRNA signal demonstrated increased GLP-1R expression in the PVNmc in post mortem hypothalamic samples from T2DM subjects as compared to controls, while there was no difference in the expression level of GLP-1R in the other subdivisions of the PVN, the hypothalamic dorsomedial and infundibular nuclei. Our results in the PVN were confirmed by RT-qPCR. Furthermore, we demonstrated by Western blot technique that the GLP-1R protein level was also elevated in the PVN of T2DM patients. GLP-1 fibre terminals were also observed in the PVNmc closely apposing oxytocin neurons using immunohistochemistry. The data suggest that GLP-1 activates GLP-1Rs in the PVNmc and that GLP-1R is elevated in T2DM patients, which may be related to the dysregulation of feeding behaviour and glucose homeostasis in T2DM.

## 1. Introduction

Glucagon-like peptide-1 (GLP-1) has received major attention in recent years. Agonists acting on its receptor and enzyme blockers preventing its degradation are used to treat type 2 Diabetes Mellitus (T2DM) patients. These drugs decrease the blood glucose level and also reduce appetite and lead to weight loss [1,2,3,4]. The 37 amino acid long peptide GLP-1 is produced primarily in L-type enteroendocrine cells of the gut in response to the ingestion of nutrients [5,6]. In addition, GLP-1, which is formed from peptide-precursor pre-pro-glucagon (PPG) by enzymatic cleavage, is also synthesized in the central nervous system. PPG mRNA expression was found in the nucleus of the solitary tract (NTS), in the dorsal and ventral medulla and the olfactory bulb of the rat brain [7]. Based on research conducted on rodents in the past decades, it became clear that PPG neurons in the NTS are the main source of endogenous central GLP-1 [8,9] including the hypothalamus [10]. GLP-1 neurons in the NTS receive direct projections from the vagal nerve fibres and are stimulated by inputs of gastrointestinal origin through these fibre terminals [11,12]. The GLP-1 neurons have ascending projections among which the hypothalamus is most extensively targeted by GLP-1 terminals [11,13,14]. Within the rodent hypothalamus, different regions, all involved in the control of food intake, contain an abundant GLP-1 fibre network, such as the paraventricular hypothalamic nucleus (PVN), the dorsomedial hypothalamic nucleus (DMH) and the arcuate nucleus [10,15,16,17,18,19], while similar distributional data on GLP-1 are missing in humans [19,20].

GLP-1 acts on a G-protein coupled receptor, called the GLP-1 receptor (GLP-1R). The expression pattern of GLP-1R in the hypothalamus is very similar to the distribution of GLP-1 fibre terminals [7,21,22,23] suggesting that GLP-1 of NTS origin exerts its actions via these receptors. In addition, peripheral GLP-1 might also reach the central GLP-1R via circumventricular organs [24,25]. A number of studies demonstrated that both the peripheral GLP-1Rs, e.g., by inducing insulin secretion from beta cells of the pancreas, and central nervous system GLP-1Rs contribute to the beneficial actions of GLP-1R agonists [24,26,27,28] including the demonstration that the food intake reducing effect of GLP-1R agonists are blunted by central infusion of GLP-1R antagonists [29]. Within the brain sites of action, the reduced food intake and the consequent loss of body weight may be primarily due to the activation of GLP-1Rs in PVN because localized intracranial injection of GLP-1R agonists into the PVN reduced food intake more dramatically than other hypothalamic injection sites in experimental animals [30,31,32]. Animal studies also demonstrated that GLP-1R in the PVN may be co-expressed by multiple cell types including oxytocin (Oxy), corticotropin-releasing hormone, nesfatin-1 and melanocortin 4 receptor-containing neurons [33,34], but any special role of these GLP-1Rs has not been established yet.

In humans, the distribution of GLP-1R in the hypothalamus was found to be similar to that in rodents, as both species showed abundant expression of GLP-1R in the PVN, the DMH and the infundibular nucleus (Inf) [35]. Since the PVN plays a role in energy balance and glucose homeostasis [36], in which its GLP-1R neurons are likely involved [34,37], we hypothesized that GLP-1R in the nucleus may be affected in type 2 diabetic patients. Therefore, we addressed this question in the present study both at the mRNA and protein levels using post mortem human brain tissue samples. To further establish whether changes in the expression of GLP-1R take place in a specific part of the hypothalamus, quantitative in situ hybridization histochemistry was also performed. In these studies, adjacent sections were labelled with Oxy to better establish the boundaries of the PVN and its subdivisions [38]. Finally, the presence of GLP-1 fibres in the PVN and their anatomical relationship to Oxy neurons were also addressed.

## 2. Results

### 2.1. GLP-1R mRNA Levels in Post Mortem Hypothalamic Samples of T2DM Patients

**Figure 1:**
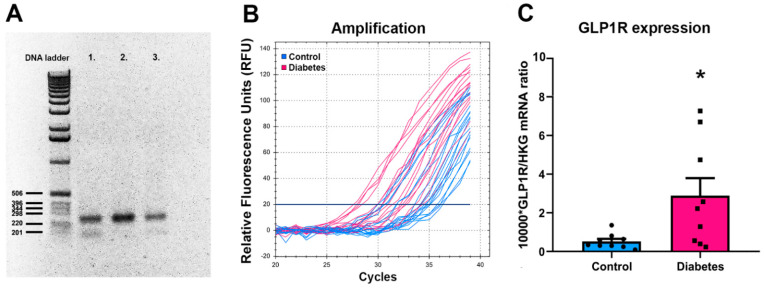
Increased level of GLP-1R in T2DM patients by qRT-PCR. (**A**) PCR products were examined by agarose gel electrophoresis. The relative gene products of GLP-1R from the PVN of T2DM samples are indicated with numbers (1, 2 and 3). The exact molecular weight of the GLP-1R product is 258 bp. (**B**) The amplification graphs demonstrate that the GLP-1R expression levels were significantly higher in the hypothalamus of T2DM diabetic individuals compared to the control group. (**C**) Quantification of the mRNA expression of GLP-1R in the hypothalamus. Bar graph represents the mean ± SEM of 9 individuals for both groups. *p*-value was calculated using the unpaired *t*-test (* *p* < 0.05).

The applied primer pairs for GLP-1R resulted in the expected molecular weight (Figure 1A), and the melting curve analysis also indicated proper product as assessed from the single peak. There were generally smaller threshold cycle values in T2DM patients for GLP-1R (Figure 1B) while the threshold cycle values for the housekeeping gene were not different. Quantitation showed that GLP-1R expression was increased in T2DM patients: the relative GLP-1R mRNA expression in the hypothalamus of diabetic individuals represented as 10,000*mRNA level/mRNA level of housekeeping genes (HKG) was 2.89 ± 0.9 in diabetics (among them, 1.39 ± 0.83 in Metformin-treated patients) and 0.52 ± 0.13 in control individuals (*p* < 0.05).

### 2.2. GLP-1R Protein Levels

**Figure 2:**
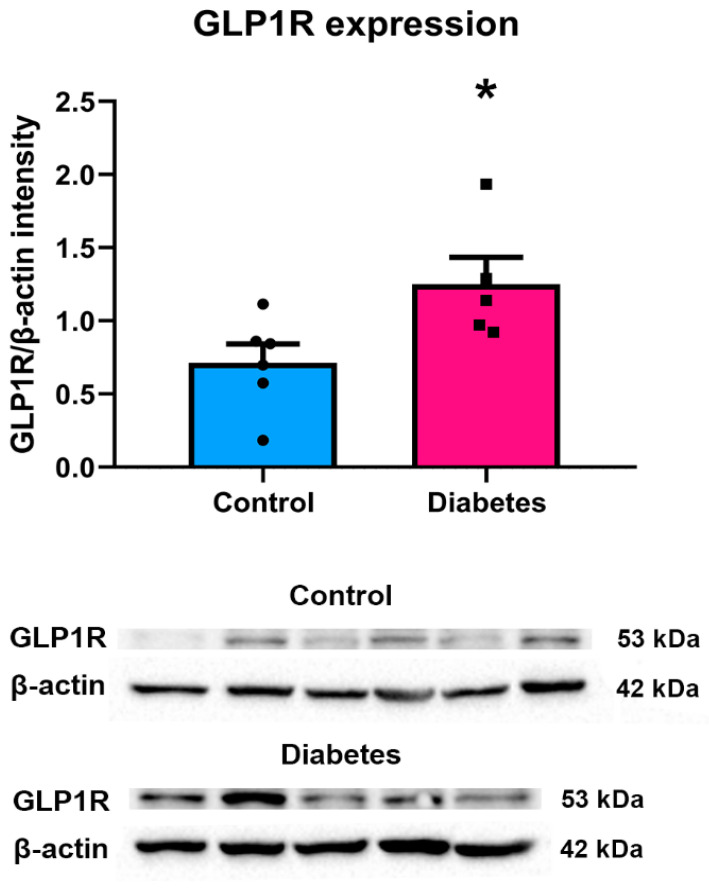
GLP-1R protein levels in the hypothalamus of T2DM patients as compared to control individuals. The hypothalamic samples were collected from the mediodorsal portion of the hypothalamus (including PVN) of six control and five T2DM diabetic individuals. Densitometry analysis showed that GLP-1R expression was significantly increased in the hypothalamus of T2DM patients. Bar graphs represent the mean ± SEM, and *p*-value was calculated using the unpaired *t*-test. (* *p* < 0.05).

We observed an increased GLP-1R protein level in the hypothalamus of diabetic individuals: 1.25 ± 0.18 in diabetics (among them, 1.45 ± 0.48 in Metformin treated patients) and 0.71 ± 0.13 in control individuals (*p* < 0.05) (Figure 2).

### 2.3. GLP-1R in the Hypothalamus

**Figure 3:**
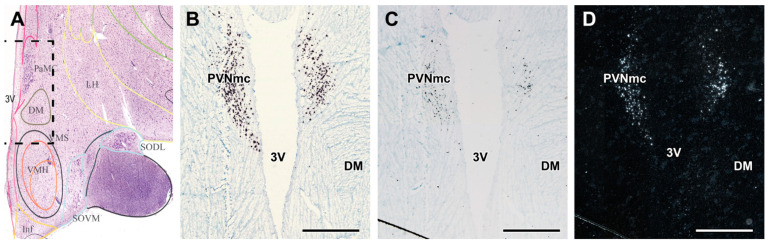
GLP-1R mRNA expression in the human hypothalamic paraventricular nucleus, magnocellular subdivision (PVNmc) in a diabetic patient. (**A**): A Nissl-stained human coronal section containing the PVNmc [39]. The dashed lined box indicates the field corresponding to the higher magnification images in (**B**–**D**). (**B**): Bright-field picture of a section containing oxytocin mRNA by in situ hybridization histochemistry. The labelled cells (black) are distributed in the PVNmc. (**C**): Bright-field picture of a section containing GLP-1R mRNA by in situ hybridization histochemistry. The labelled cells (black) have a similar distribution as the oxytocin neurons in the adjacent section shown in panel B. Note that autoradiography was performed for Oxy only for 1 day as opposed to 3 weeks for GLP-1R. (**D**): A dark-field image of the same field presented in panel C shows better visualization of GLP-1R-expressing neurons. Additional abbreviations: DM—dorsomedial hypothalamic nucleus, 3V—third ventricle. Scale bars = 2 mm.

Labelled neurons were characterized by an accumulation of autoradiography grains above the cells. We observed GLP-1R-expressing in distinct parts of the hypothalamus including the PVN, the infundibular nucleus and the dorsomedial hypothalamic nucleus. Labelling of individual cells seemed similarly intensive in these brain regions; however, the density of labelled neurons was the highest in the PVN. The precise localization of the labelled cells was determined by strong labelling of oxytocin in the magnocellular subdivision of the PVN (PVNmc) (Figure 3). The much longer autoradiography procedure required for GLP-1R suggests a lower expression level of the receptor than for Oxy.

### 2.4. GLP-1R Level in T2DM Patients in Various Parts of the Hypothalamus

**Figure 4:**
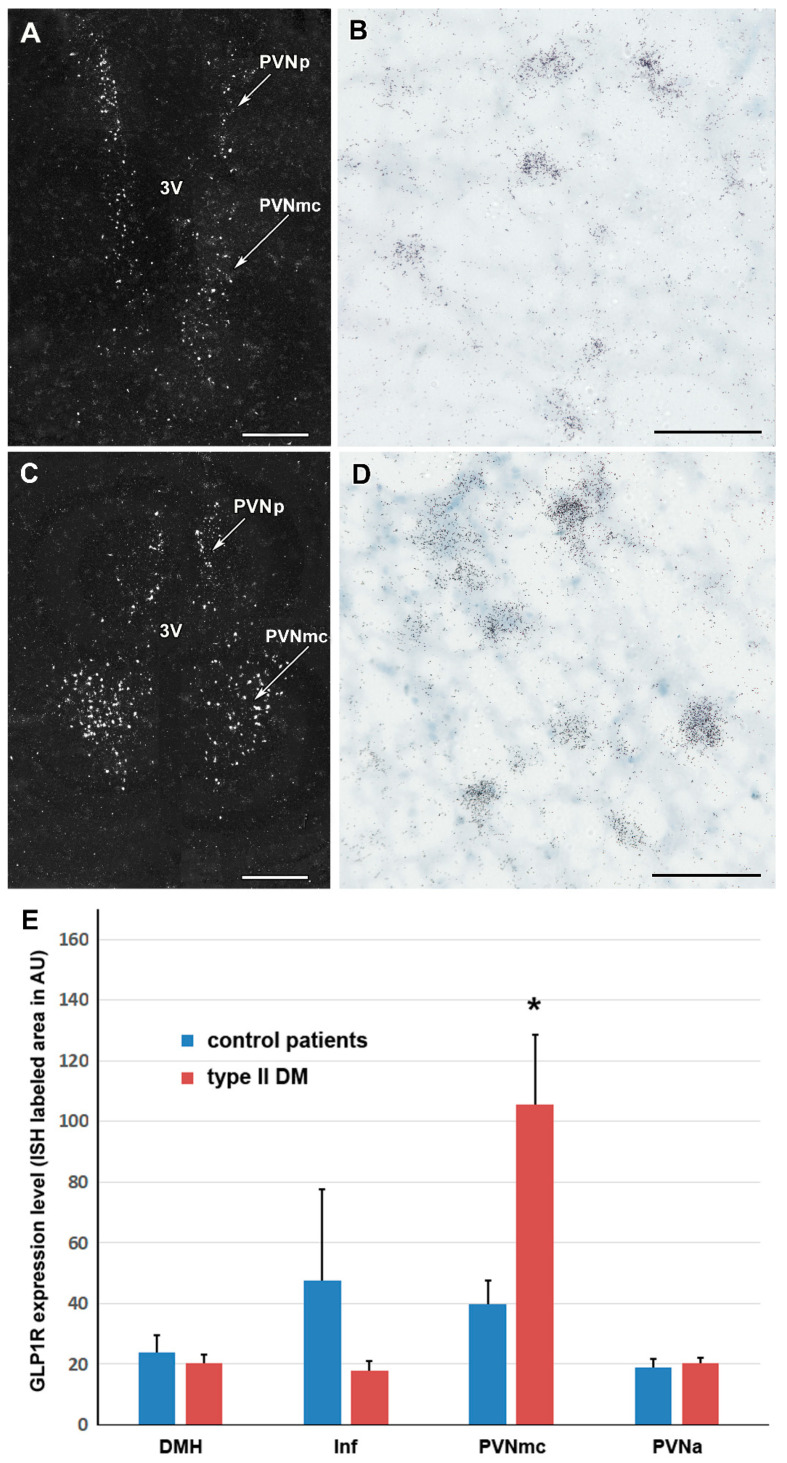
Images of in situ hybridization histochemistry sections show the expression of GLP-1 receptor in the medial hypothalamus of a control patient. (**A**): A dark-field image demonstrates GLP-1 receptor expression in the parvicellular (PVNp) and magnocellular part of the paraventricular nucleus (PVNmc) of a non-diabetic control patient. (**B**): The PVN is shown in a higher magnification bright-field image where labelled cells appear as the accumulation of small black dots representing individual autoradiography grains. (**C**,**D**): Images of in situ hybridization histochemistry sections show the expression of GLP-1R in the medial hypothalamus of a type 2 diabetic patient. (**C**): A dark-field image demonstrates GLP-1R expression in the parvicellular (PVNp) and magnocellular paraventricular nucleus (PVNmc) of a diabetic patient. (**D**): The PVNmc is shown in a higher magnification bright-field image. (**E**): The density of GLP-1 receptor mRNA in the dorsomedial hypothalamic (DMH), infundibular (Inf), paraventricular hypothalamic, magnocellular (PVNmc) and paraventricular hypothalamic nucleus, anterior (parvocellular) subdivision (PVNa) in control (n = 5) and type 2 diabetic patients (n = 5). The density of the autoradiographic signal was calculated with Image J software as the number of labelled pixels is expressed in arbitrary units (AU). *: *p* < 0.05. Additional abbreviations: 3V—third ventricle. Scale bars = 1 mm for (**A**,**C**), and 100 µm for (**B**,**D**).

GLP-1R mRNA level was measured as the number of white (above threshold) pixels due to labelling with autoradiography grains in dark-field 40X images taken randomly from the particular hypothalamic nuclei. We found a markedly increased GLP-1R level in the PVNmc of T2DM patients as compared to controls while no difference was found in the expression level of GLP-1R in the anterior parvocellular subdivision of the PVN, the infundibular and dorsomedial hypothalamic nuclei (Figure 4).

### 2.5. GLP1 Fibres in the PVN

**Figure 5:**
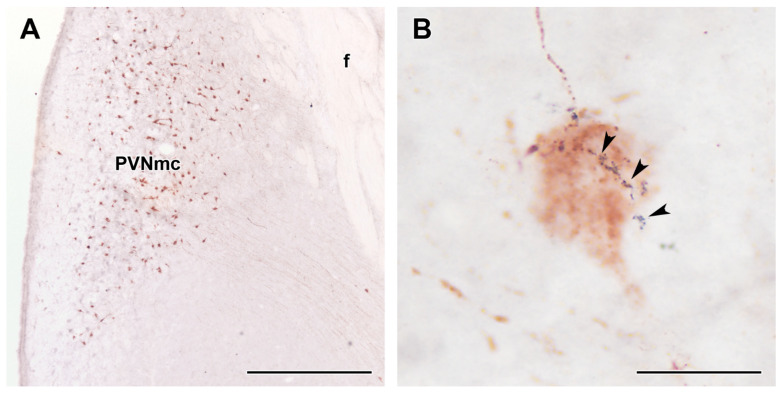
GLP-1 fibre terminals (black) approach oxytocin cell bodies (brown). The double labelling was performed using the DAB-Ni-DAB method. (**A**): Oxytocin neurons are distributed in the PVN, especially its magnocellular subdivision (PVNmc). (**B**): A high magnification image to show an example of oxytocin neuron closely apposed by a GLP-1 fibre forming varicosities (black arrows). Additional abbreviation: f = fornix. Scale bars = 1 mm for (**A**) and 25 µm for (**B**).

A GLP-1-immunoreactive signal was present in the PVN of the hypothalamus, especially its magnocellular subdivision where Oxy neurons were also located (Figure 5A). The signal was present in neuronal fibres as assessed by its appearance in consecutive varicosities. These varicosities often approached Oxy neurons forming close appositions between fibre terminals and cell bodies of Oxy neurons (Figure 5B).

## 3. Discussion

### 3.1. The Presence of the GLP-1 System in the Human Hypothalamus

The presence of GLP-1 peptide in the human PVN was first demonstrated in our study. We also showed the presence of GLP-1R mRNA in the human hypothalamus confirming previous findings [40]. The distribution determined by in situ hybridization histochemistry is in line with previous rodent [7,21] as well as human studies [35]. We further showed the presence of GLP-1R protein in the human hypothalamus by Western blotting, which represents the first such finding to our knowledge.

### 3.2. Elevated GLP-1R in T2DM Patients

The GLP-1R mRNA level was increased in the hypothalamus of T2DM patients using two independent techniques at the mRNA level. This finding contradicts a previous study, which detected a reduced level of GLP-1R mRNA in the hypothalamus using a single technique, in situ hybridization histochemistry [35]. A reason for this discrepancy could be differently sampled brain areas as both our and the previous study examined only a selected part of the hypothalamus, which is partially but not entirely overlapping. Another possibility is that T2DM patients with different clinical histories were used in our and the previous study. To further address this issue, we also measured GLP-1R at the protein level. The elevated amount of GLP-1R protein in our samples suggest that the GLP-1R function is potentially enhanced in T2DM patients.

Medication before death, post mortem delay of freezing the brains and several other factors could affect GLP-1R mRNA level. Most importantly, Metformin can cause increase in the level of GLP-1R protein as demonstrated previously in human umbilical vein endothelial cell culture [41]. In addition, Metformin also increased the level of GLP-1R mRNA in pancreatic islets of mice [42]. Since there is strong evidence that metformin can cross the blood–brain barrier to act in the central nervous system [43,44], it is possible that direct action of metformin on PVN neurons can contribute to the elevated GLP-1R levels. Based on our data, however, it is not likely because the metformin treated patients had a lower mRNA and similar protein level of GLP-1R than the non-metformin treated patients. It is also important to note that no effects of sulfonylurea derivatives on GLP-1R expression have been reported in the literature to our knowledge.

In situ hybridization histochemistry suggested that the increase in GLP-1R level is not ubiquitous in the hypothalamus of T2DM patients. An increased expression was found in the magnocellular subdivision of the PVN (PVNmc), but not in the anterior subdivision of the PVN, the DMH and Inf. These findings suggest that the increase in whole hypothalamic samples used in RT-qPCR was due to increased GLP-1R expression in the PVNmc. The spatially restricted increase of GLP-1R also suggests an outstanding function of GLP-1R in the PVNmc. While our own data are insufficient to draw conclusions, the PVN is the site where GLP-1 can exert the strongest effect on food intake [30,31]. An elevated level of GLP-1R in this region could be a compensatory mechanism in overweight T2DM patients. Our data also provide no information on how GLP-1R mRNA is increased in T2DM patients. It has been argued that alterations in the GLP-1 system are not the reason but the consequence of other events taking place in T2DM patients [45].

### 3.3. Functional Implications of the PVN GLP-1 System

While we cannot exclude the possibility that peripheral GLP-1 acts on GLP-1Rs in the PVNmc, it is more likely that GLP-1 of brain origin activates PNVmc neurons [46]. To address this issue, we described GLP-1-containing fibres in the PVNmc. Since in rodents [8], as well as macaques [19], direct projections of NTS GLP-1 neurons to the PVN were shown, we can assume that these fibres originate in the NTS in the human brain as well. We found close apposition between GLP-1 fibre terminals and oxytocin cell bodies in the PVNmc suggesting that GLP-1 may act on Oxy neurons. Since Oxy neurons have well known anorexigenic effects [47] to which GLP-1 can contribute in rodents [48], it is plausible that GLP-1 exerts its effect on Oxy neurons of the PVNmc. However, we also have to emphasize that our data did not prove the presence of GLP-1R in Oxy neurons, which therefore remains an open question. In rodents, GLP-1R has been demonstrated in Oxy neurons of the PVN but also in other types of PVN neurons suggesting that multiple cell types mediate the effect of GLP-1 [33]. Similarly, the beneficial effects of GLP-1R agonists [49] may be mediated by multiple types of neurons in T2DM patients. Based on our data on the elevated GLP-1R in the PVNmc, it is likely, however, that their effectiveness within the PVNmc is elevated, which could contribute to their usefulness in T2DM.

## 4. Materials and Methods

### 4.1. Human Brain Tissue Samples

Human brain samples were collected in accordance with the Ethical Rules for Using Human Tissues for Medical Research in Hungary (HM 34/1999) and the Code of Ethics of the World Medical Association (Declaration of Helsinki). Tissue samples were taken during brain autopsy at the Department of Pathology, Forensic and Insurance Medicine of Semmelweis University and at the Clinical Centre of Pathology, University of Debrecen in the framework of the Human Brain Tissue Bank (HBTB), Budapest. The activity of the HBTB has been authorized by the Committee of Science and Research Ethic of the Ministry of Health Hungary (ETT TUKEB: 189/KO/02.6008/2002/ETT) and the Semmelweis University Regional Committee of Science and Research Ethic (No. 32/1992/TUKEB). The present study was performed according to a protocol approved by the Committee of Science and Research Ethics, Semmelweis University (TUKEB 189/2015). The medical history of the subjects was obtained from clinical records, interviews with family members and relatives, as well as pathological and neuropathological reports (Table 1). All personal data are stored in strict ethical control, and samples were coded before the analyses of tissue.

### 4.2. Tissue Preparation

**Figure 6:**
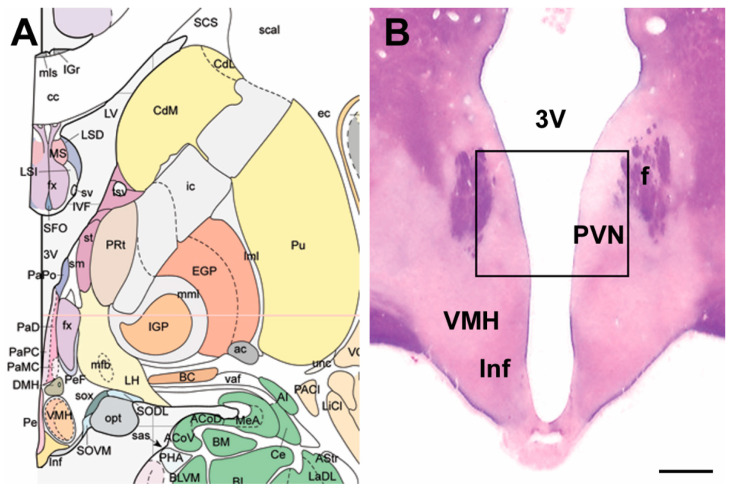
A representative view of the human brain region dissected in the study. (**A**) The location and topographical extension of the brain area [39]. (**B**) Coronal section of the human hypothalamus containing the dissected area. The dissected area analysed in this study is demarcated by a black box. The section was stained using the Levanol-Fast Cyanine 5RN method. The scale bar is 2 mm. Abbreviations: f—fornix, Inf—infundibular nucleus, PVN—paraventricular hypothalamic nucleus, VMH—ventromedial hypothalamic nucleus.

Post mortem human brain tissue samples from the hypothalamus were acquired from the Human Brain Tissue Bank (Semmelweis University, Budapest, Hungary). The dissected hypothalamic regions corresponded to the paraventricular hypothalamic nucleus (Figure 6).

The hypothalamic samples were collected from 18 subjects: 9 control subjects with no history of diabetes (4 females and 5 males, mean age of 71.1 ± 3.8) and 9 patients with T2DM (4 females and 5 males, mean age of 77.4 ± 2.6). The age of the subjects ranged from 52 to 95 years (Table 1). The selected control subjects were not diagnosed with diabetes. T2DM patients whose brain samples were included in the study were either not treated, or biguanid-type antihyperglicemic agent metformin (Merckformin or Meforal) or sulphonylureas, such as gliclazide (Gliclada or Diaprel) were prescribed for them. In turn, none of the T2DM patients received GLP-1R agonist treatment. Brains were removed from the skull with a post mortem delay of 1 to 10 h, frozen rapidly on dry ice and stored at −80 °C until microdissection. Serial coronal sections were cut from the diencephalon. Sections 4.0 mm from the origin of AC-PC coordinates were punched out (see [39]). Special microdissection needles with 1 mm inside diameters were used [50,51]. Tissue pellets (1–3 per brain) include samples from the hypothalamus. The microdissected tissue pellets were collected in 1.5 mL Eppendorf tubes and kept until use at −80 °C. During each step of the microdissection procedure (slicing, micropunch, storage), the tissue samples were kept frozen.

### 4.3. Gene Expressional Measurements with qRT-PCR

The procedure was carried out as described previously [52]. Briefly, total RNA was isolated from approximately 20 mg of frozen post mortem brain tissue using TRIzol reagent (Invitrogen, Carlsbad, CA, USA) as lysis buffer combined with RNeasy Mini kit (Qiagen, Germany) following the manufacturer’s instructions. The quality and quantity of extracted RNA were determined using NanoDrop ND-1000 Spectrophotometer (Thermo Fisher Scientific, Waltham, MA, USA), and only those with A260/A280 ratio between 1.8 and 2.1 were used in subsequent experiments. The concentration of RNA was adjusted to 1000 ng/µL, and it was treated with Amplification Grade DNase I (Invitrogen, Carlsbad, CA, USA). The isolated RNA concentration was calculated and normalized with RNase-free water and reverse transcribed into cDNA using SuperScript II Reverse Transcriptase Kit (Invitrogen, Carlsbad, CA, USA). After 10-fold dilution, 2.5 μL of the resulting cDNA was used as a template in PCR performed in duplicates using SYBR Green dye (Sigma, St. Louis, MO, USA). The PCR reactions were performed on CFX-96 C1000 Touch Real-Time System (Bio-Rad Laboratories, Hercules, CA, USA) with iTaq DNA polymerase (Bio-Rad Laboratories, Hercules, CA, USA) in total volumes of 12.5 μL under the following conditions: 95 °C for 3 min, followed by 35 cycles of 95 °C for 0.5 min, 60 °C for 0.5 min and 72 °C for 1 min. A melting curve was performed at the end of amplification cycles to verify the specificity of the PCR products. The primers used for qRT-PCR were synthesized by Integrated DNA Technologies, Inc., (IDT, Coralville, IA, USA) and used at 300 nM final concentration. Housekeeping genes ACTB, GAPDH and LDHA were used as internal controls. The applied primers were CCCCAGACACATTCTCCTGT and AGCAAATAAGGGGTGCCTTT for GLP-1R, GTGCTATCCCTGTACGCCTC and TGGCCATCTCTTGCTCGAAG for β-actin, GGAGGTTGTGCATGTTGTCC and CAGTGAAGGAGCCAGGAAGT for LDHA, and CCAGAACATCATCCCTGC and GTGGGTGTCGCTGTTGAA for GAPDH. The relative gene expression values were calculated from the ratio of GLP-1R and the averages of housekeeping genes using the 2^−△△Ct^ method.

### 4.4. Protein Extraction for Western Blotting

Proteins were isolated from the stored fraction remaining after RNA extraction as described previously [53]. Briefly, after DNA precipitation, isopropanol was added to the supernatant to precipitate protein. The protein precipitate was washed twice with 0.3 M guanidine hydrochloride in 95% ethanol, then the protein precipitate was vacuum-dried and dissolved in 1% SDS mixed with protease inhibitors. The protein concentration was determined using BCA kit (Sigma-Aldrich, cat. no. BCA1-1KT), where bovine serum albumin was used as a standard.

### 4.5. Western Blot Analysis

Protein extracts of the PVN were obtained for Western blotting. A measure of 20 μg protein was subjected to standard SDS-PAGE using 15% polyacrylamide gels and transferred to nitrocellulose (Bio-Rad, Cat. No. 1620112). The membrane was blocked with 5% non-fat dry milk diluted in Tris-buffered saline-Tween (TBS-T) buffer (20 mM Tris, 150 mM NaCl, 0.1% Tween-20, pH 7.6). Anti-GLP-1R antibody (mouse anti-GLP-1, Santa Cruz Biotechnology, Cat. No. number. sc-57166) at a ratio of 1:500 dissolved in 5% BSA in in TBS-T and anti-β-actin antibody (Sigma-Aldrich, Cat. No. A2228) at a ratio of 1:10.000 dissolved in 5% non-fat dry milk were applied and incubated overnight at 4 °C. The bound anti-GLP-1R antibodies were labelled with anti-rabbit (1:2000; Jackson ImmunoResearch, Cat. No. 711035152) and anti-β-actin antibodies were labelled with anti-mouse (1:2000; Jackson ImmunoResearch, Cat. No. 715035150) horseradish peroxidase-conjugated secondary antibody followed by visualization with Gel Doc XR+ imaging system (BioRad) using Clarity Western ECL Substrate (BioRad Laboratories, Cat. No. 170–5060). The molecular weight of the labelled proteins was measured with Spectra™ Multicolour High Range Protein Ladder (Cat. No. 26625, Thermo Scientific, Waltham, MA, USA).

### 4.6. Preparation of In Situ Hybridization Probes

A mixture of cDNAs prepared with RT-PCR from the human hypothalamus was used as a template in PCR reactions. For GLP-1 (accession number: NM_002062.5), the following primers were used: GACAGGTAAATGGGCAGTGC and GTGACCCCAAGTGATGCAAG. For oxytocin (accession number: M25650.1), the following primers were used: GCTGGACGCCTTTCTTCTTC and CAGGACAAAGGAGGACGAGT). The purified PCR products were applied as templates in a PCR reaction with the primer pairs specific for the probe and also containing the T7 RNA polymerase recognition site added to the reverse primers. Finally, the identities of the cDNA probes were verified by sequencing them with T7 primers.

### 4.7. In Situ Hybridization Histochemistry

Eleven fresh-frozen hypothalamic brain blocks of subjects were used. Using a cryostat, serial coronal sections (12 μm) were cut and mounted on positively-charged slides (SuperfrostPlus^®^, Fisher Scientific), dried and stored at −80 °C until use. Further steps were performed according to the procedure described previously [54]. Briefly, antisense [35S] UTP-labelled riboprobes were generated from the above-described DNA probes using T7 RNA polymerase of the MAXIscript Transcription Kit (Ambion, Austin, TX, USA) and used for hybridization at 1 million DPM (discharges per minute) activity per slide. Washing procedures included a 30 min incubation in RNase A followed by decreasing concentrations of sodium-citrate buffer (pH 7.4) at room temperature and subsequently at 65 °C. Following hybridization and washes, slides were dipped in NTB nuclear track emulsion (Eastman Kodak) and stored at 4 °C for 3 weeks (GLP-1R), or 1 day (Oxy) for autoradiography. Then, the slides were developed and fixed with Kodak Dektol developer and Kodak fixer, respectively, counterstained with Giemsa and coverslipped with Cytoseal 60 (Stephens Scientific, Kalamazoo, MI, USA). The density of autoradiographic signal was calculated with Image J software following microscopic photography in the dark-field using 40X objective.

### 4.8. Tissue Collection for Immunolabelling

Immunohistochemistry was used to assess the distribution of GLP-1 and oxytocin in the hypothalamus. For immunolabelling, a hypothalamus block from a 79-year-old female control individual was cut into 50 mm thick coronal slice and immersion fixed in 4% paraformaldehyde in 0.1 M phosphate-buffered saline (PBS) for 5 days. Subsequently, the block was transferred to PBS containing 0.1% sodium azide for 2 days to remove excess paraformaldehyde. Then, the block was placed in PBS containing 20% sucrose for 2 days for cryoprotection. The block was frozen and cut into 60 μm thick serial coronal sections on a sliding microtome. Sections were collected in PBS containing 0.1% sodium azide and stored at 4 °C until further processing.

### 4.9. Immunolabelling

Free-floating hypothalamic sections were immunolabelled for GLP-1 and oxytocin using the following primary antibodies: (mouse anti-GLP-1, Santa Cruz Biotechnology, Cat. No. number sc-57166 and mouse anti-Oxy, Merck, Cat. No. MAB5296). The anti-GLP-1 antibody (1:100 dilution) and the anti-Oxy (1:2000 dilution) antibodies were applied for 24 h at room temperature, followed by incubation of the sections in biotinylated anti-mouse secondary antibody (1:1000 dilution, Vector Laboratories, Burlingame, CA, USA) and then in avidin-biotin-peroxidase complex (1:500, Vector Laboratories) for 2 h. Subsequently, the labelling was visualized by incubation in 0.02% 3,3-diaminobenzidine (DAB; Sigma), 0.08% nickel (II) sulphate and 0.001% hydrogen peroxide in PBS, pH 7.4 for 5 min. Sections were mounted, dehydrated and coverslipped with Cytoseal 60 (Stephens Scientific, Riverdale, NJ, USA).

### 4.10. Double Labelling of GLP-1 and Oxytocin

Double immunolabelling was used to establish the precise anatomical relationship between GLP-1 terminals and oxytocin neurons. Slides were blocked by incubation in 3% bovine serum albumin (with 0.5% Triton X-100 dissolved in 0.1 M PB, Sigma) for 1 h at room temperature, followed by washing with washing buffer (10 min × 3). GLP-1 was immunolabelled as for single labelling. Subsequently, sections were placed in mouse anti-oxytocin antibody) (1:500 dilution, Merck, Cat. No. MAB5296) for 24 h at room temperature. The sections were then incubated in biotinylated donkey anti-mouse secondary antibody (1:500 dilution, Vector Laboratories) followed by 2 h incubation in a solution containing avidin-biotin-peroxidase complex (ABC, 1:300 dilution, Vector Laboratories). Finally, all sections were visualized without nickel (II) sulphate with the following parameters: incubation in 0.05% 3,3-diaminobenzidine (DAB; Sigma) and 0.002% hydrogen peroxide in PBS, pH 7.4 for 8 min. Sections were mounted, dehydrated and coverslipped with Cytoseal 60 (Stephens Scientific, Riverdale, NJ, USA).

### 4.11. Microscopy and Photography

Sections were examined using an Olympus BX60 light microscope also equipped with fluorescent epi-illumination and a dark-field condenser. Images were captured at 2048 × 2048-pixel resolution with a SPOT Xplorer digital CCD camera (Diagnostic Instruments, Sterling Heights, MI, USA) using a 4× objective for dark-field images, and 4–40× objectives for bright-field and fluorescent images. Contrast and sharpness of the images were adjusted using the ‘levels’ and ‘sharpness’ commands in Adobe Photoshop CS 8.0. Full resolution was maintained until the photomicrographs were cropped at which point the images were adjusted to a resolution of at least 300 dpi.

### 4.12. Statistical Analysis

Demographics were contrasted using the Chi-Square test or Welch’s unequal variances *t*-test (*p* < 0.05). Quantitative statistics of RT-qPCR results were performed with GraphPad Prism version 8.0.1 (GraphPad Software). For comparisons between values of the two groups, unpaired *t*-test was applied. Differences were considered statistically significant when *p* < 0.05 and plotted as mean ± S.E.M (standard error of the mean).

## 5. Conclusions

We performed a post mortem gene expressional study of the hypothalamus in patients of type 2 Diabetes Mellitus, using RT-qPCR, Western blotting, in situ hybridization histochemistry and immunohistochemistry and observed the presence of GLP-1 as well as GLP-1 receptor (GLP-1R) in the paraventricular hypothalamic nucleus (PVN). In addition, we first report the induction of GLP-1R in the human PVN in diabetic patients. The presented findings that GLP-1 receptor is abundant in the magnocellular subdivision of the PVN (PVNmc) and is overexpressed in type 2 diabetes mellitus (T2DM) suggest that GLP-1R in the PVNmc may have special role in the dysregulation of feeding behaviour and glucose homeostasis in T2DM. The results also suggest that GLP-1R is located in oxytocin neurons of the PVNmc. Furthermore, GLP-1 fibres arising to the PVNmc from the nucleus of the solitary tract may be the targets of ascending GLP-1-containing neurons, which at least in the rat, transmit information on satiety. The markedly increased level of GLP-1 receptor in type 2 diabetes suggests altered processing of the satiating signal in these persons. GLP-1 may act on its receptor to affect the activity of oxytocin cells potentially influencing mood and social activity. The elevated level of GLP-1R might be a consequence of elevated insulin level in T2DM. We conclude that our study provides new insights into the pathophysiology of T2DM.

## Figures and Tables

**Table 1:** Relevant data of individuals.

Donor #	Experimental Group	Age	Sex	Clinical and Pathological Diagnosis (in Addition to T2DM in the Experimental Group)	Cause of Death	Post Mortem Interval/Hour
1	Control	72	female	Hypertonia	Acute cardiac insufficiency	4.5
2	Control	67	male	Hypertonia	Pulmonary embolism	5
3	Control	81	female	General atherosclerosis	SStroke (brain haemorrhage)	5
4	Control	80	male	Hypertonia, coronary sclerosis	Stroke (acute intracerebral haematoma)	2
5	Control	70	female	Hypertonia	Stroke	7
6	Control	65	male	Hypertonia, coronary sclerosis	Stroke, broncho-pneumonia	10
7	Control	95	female	Hypertonia, arteriosclerosis	Stroke	4
8	Control	52	male	Chronic alcoholism	No information	8
9	Control	66	male	Cardiorespiratory insufficiency	Stroke	9
10	T2DM	69	female	Pulmonary insufficiency, hypertonia	Stroke	3
11	T2DM	67	male	Duodenal ulcer	Pneumonia, cardiopulmonary insufficiency	10
12	T2DM	75	female	General atherosclerosis, mamma carcinoma	Stroke	10
13	T2DM	79	male	Hypertonia, cardiomyopathia	Stroke	6
14	T2DM	92	female	Cardiorespiratory insufficiency, arteriosclerosis universalis, renal insufficiency, hypertonia	oOedema cerebri	4
15	T2DM	74	male	Hypertonia, prostate carcinoma	Stroke, oedema cerebri	3
16	T2DM	85	female	Hypertonia, arteriosclerosis	Stroke	8
17	T2DM	79	male	Obesity, polyneuropathia	Cardiorespiratory insufficiency, stroke	10
18	T2DM	77	male	Chronic alcoholism, cardiorespiratory insufficiency	Stroke, bronchopneumonia	5.5

## Data Availability

The data not presented in the paper are available from the corresponding author upon request.
